# Testing the effectiveness of user-tested patient information on recruitment rates across multiple trials: meta-analysis of data from the start programme

**DOI:** 10.1186/1745-6215-16-S2-P84

**Published:** 2015-11-16

**Authors:** Peter Bower, Jo Rick, Anne Kennedy, Chris Salisbury, David Collier, David Torgerson, Jonathan Graffy, Shaun Treweek, Sandra Eldridge, Vichithranie Madurasinghe, Peter Knapp, Adwoa Hughes-Morley, Nicola Small

**Affiliations:** 1 University of Manchester, Manchester, UK; 2 University of York, York, UK; 3 Queen Mary's University of London, London, UK; 4 University of Bristol, Bristol, UK; 5 University of Cambridge, Cambridge, UK; 6 University of Aberdeen, Aberdeen, UK; 7 University of Southampton, Southampton, UK; 8 University of Dundee, Dundee, UK

## Background

The evidence base for trial recruitment is minimal. The START research programme aims to test interventions to improve recruitment by embedding recruitment interventions across multiple ‘host’ trials.

Patient understanding of study information is critical to informed consent, but there is concern that patient information materials can be complex and deter potential participants. User testing may be a way of improving materials and enhancing recruitment.

Our aim was to test the effects of user-tested patient information materials on recruitment rates across multiple trials.

## Methods

We embedded trials of the optimised materials across multiple ongoing ‘host’ trials.

Patients identified as potentially eligible in each of the trials were randomised to receive either the original patient information materials or optimised, user-tested versions.

Primary outcomes were the proportion of participants randomised .

## Results

At present, 8 trials have taken part and 4 trials have completed. The current analysis across all trials suggests that optimised materials have a modest and non-significant impact on randomisation (RR 1.08, 95% CI 0.95 to 1.23, I squared=0).

At the conference, we will present the most up-to-date results from the ongoing meta-analysis.

## Conclusions

Recruitment to trials is not underpinned by a strong evidence-base. Embedding trials of recruitment interventions across multiple host studies provides a model for rapid improvement. We discuss the impact of the enhanced materials, and insights on the barriers to embedding recruitment trials.

